# Maternal Folic Acid-Containing Supplement Use in Relation to Offspring Motor Function. A Prospective Study of 503 Mother-Child Dyads

**DOI:** 10.3389/fped.2022.789158

**Published:** 2022-04-05

**Authors:** Kine Melfald Tveten, Roy Miodini Nilsen, Tove Dragesund

**Affiliations:** Department of Health and Functioning, Western Norway University of Applied Sciences, Bergen, Norway

**Keywords:** folic acid, multivitamin, motor function, medical birth registry, pediatric epidemiology, maternal health

## Abstract

**Background:**

The preventive effect of maternal folic acid use on offspring neural tube defects is well-established. However, a putative link between supplement use and other neurodevelopmental outcome is inconsistent. The aim of this study was to examine the association of folic acid-containing supplement use before and during pregnancy with motor function in children aged 3–18 months.

**Method:**

The study has a prospective cohort design including 503 mother-infant dyads. Motor function was measured by the Infant Motor Profile (IMP) and Ages and Stages Questionnaire Second Edition (ASQ-2). Associations between exposure and outcome were examined using linear regression analysis with robust standard error estimation.

**Results:**

Offspring total IMP score was not associated with any maternal folic acid-containing supplement use when they were used during pregnancy only (adjusted β = 0.11 95% CI = −1.19, 1.40; *p* = 0.87) or when they were used both before and during pregnancy (adjusted β = 0.22 95% CI = −0.95, 1.40; *p* = 0.70). When examining the five domain scores separately, only the IMP domain adaptability showed some association with supplement use during pregnancy (adjusted β = 2.87; 95% CI = 0.08, 5.68; *p* = 0.04), but the strength of the association was weak. Further, supplement use was not associated with any of the two motor domains of ASQ-2.

**Conclusion:**

Although no association between folic acid-containing supplement use and offspring motor function was found, the complexity of this topic and its potential mechanisms, requires further investigation. This research should include robust and accurate measures on maternal nutritional status along with thorough endpoint assessments.

## Introduction

Maternal nutrition is vital to fetal brain development (1, 2). Among the many central micronutrients in this regard is vitamin B9 (folate), which in its synthetic form is known as folic acid. Due to the rapid neuronal proliferation during pregnancy, the achievement of sufficient folate levels through a regular diet is challenging. In Norway, there is no mandatory fortification of food products with folate. Hence, daily use of folic acid tablets or multivitamins containing folic acid is recommended to women 1 month before planned pregnancy and until 12 weeks of gestation (3). In Norway, folic acid tablets contain 400 μg and multivitamins contain 200 μg folic acid (4). The current percentage of women in Norway taking folic acid supplements before pregnancy is 39.2% and during pregnancy is 83.8%. Regarding multivitamin supplements, the percentage of women taking multivitamins before pregnancy is 22.4%, and during pregnancy is 58.1% (5).

The recommendation of maternal folic acid use is primarily built on evidence for the prevention of neural tube defects in infants (6), but folate appears to have preventive effects also on other adverse pregnancy outcomes (7–9). The need for folate increases during pregnancy because of increased demands from the fetus and rapid neuronal proliferation (10, 11). If folate deficiency during early pregnancy can cause neural tube defects, it may also cause milder forms of fetal neural impairments that could be expressed as decreased motor function (9). A possible mechanism is the level of myelinization, which has been proposed as an expression of the functional maturity of the brain and thus linked to motor development (12). However, maternal folate status during pregnancy and its impact on offspring motor function remains inconsistent or insufficiently examined (8, 9).

A positive relationship between maternal folic acid supplement use (either as folic acid tablets or folic acid-containing multivitamin supplements) and high motor scores was found in a population of Indian infants at age 2 years (*n* = 123) (13), Spanish children at age 4 years (*n* = 420) (14) and African-American infants at age 3 years (*n* = 6,774) (15). In contrast, no association between maternal folic acid-containing supplement use and offspring motor function was found in a sample of Polish infants (*n* = 538) at age 2 years (16) and a sample of American children from a low-socioeconomic family at age 5 years (*n* = 533) (17). Finally, an inverse relationship between maternal folic acid-containing supplement use and fine motor function but not gross motor function at age 2 years was found in a sample of Chinese mother-child dyads (*n* = 180) (18). A limitation in previous studies is the use of global screening instruments as outcome measures and not thorough clinical assessments.

Due to the divergent findings from previous research; the aim of the present study was to examine maternal folic acid-containing supplement use before and during pregnancy and its association with both clinical assessments and parent reports on infant motor function. Our predefined hypothesis was that infants born by mothers who did take folic acid-containing supplements (either as folic acid tablets or folic acid-containing multivitamins or both) either before or during pregnancy would be associated with higher scores in motor function compared to infants of mothers who did not use any of these supplements in pregnancy.

## Materials and Methods

We conducted a prospective, observational study of infants aged 3–18 months. Infants were recruited by public health nurses during regular well-baby check-ups in primary care in Norway. Recruitment took place in four different municipalities; Porsgrunn (36 448 residents, 309 births, 1 well-baby center), Bamble, (14,046 residents, 129 births, 1 well-baby center), Tønsberg (56 760 residents, 439 births, two well-baby centers), and Bergen (285 070 residents, 3,192 births, 17 well-baby centers) (19) in the period of 2017–2020. All infants aged 3–18 months were eligible for inclusion. Excluded were infants with parents not speaking and understanding a Scandinavian or English language.

Clinical assessments of motor function according to the Infant Motor Profile (IMP) (20) were standardized for all participants and conducted at the public health care centers or in the infant's home by a specialist in pediatric physiotherapy (KMT). Assessments were video-recorded and scored shortly after the assessment. Parents filled out the Ages and Stages Questionnaire Second Edition (ASQ-2) (21) directly after the assessment.

The Regional Committee for Medical and Health Research Ethics approved the project (2016/566 REK Vest). Mothers received a written invitation and signed informed consent before enrolment. The study followed recommendations and the checklist by the Strengthening the Reporting of Observational Studies in Epidemiology Statement (STROBE).

### Exposure

To obtain information on infant and maternal medical history, data on IMP and ASQ-2 were linked to the Medical Birth Registry of Norway (MBRN) by using the mothers' national identity number. MBRN is a national health registry that has collected information on pregnancies, births, and maternal and child health in Norway since 1967. Data on maternal characteristics before and during pregnancy are collected through standardized notification forms by the attending health care personnel during delivery (22). This notification form also includes data on supplement type (pure folic acid tablets and/or multivitamins tablets) as well as the timing of use (before and/or during pregnancy). Data on supplement use are reported by the pregnant woman and health care personnel in connection with routine pregnancy health check-ups. All multivitamins available in Norway contain folic acid (4). Hence, we defined folic acid use as any supplemental use of folic acid and/or multivitamin and labeled the exposure “folic acid-containing supplements”. Due to the different doses of folic acid in multivitamins and folic acid tablets, we also categorized the women by supplement type (i.e., multivitamin alone, folic acid alone, and both folic acid and multivitamin).

Information on the exact timing of initiation, frequency, or duration of supplement use was not available.

### Outcome

The outcome variables were assessed once when infants were between 3 and 18 months old with the clinical tool IMP and the parent-reported ASQ-2. The IMP is a clinical tool with norm references from 1,700 Dutch infants (20). IMP consists of a total score and five domain scores: variation, adaptability, symmetry, fluency, and performance. Variation is defined as the presence of a broad repertoire of motor behavior, while adaptability is the ability to select the best fitting motor strategy in each situation (23). The IMP domain adaptability is only calculated for infants >6 months in line with the IMP manual (20). The domain symmetry indicates whether the infant has a prevailing asymmetry, or if asymmetric, stereotyped postures are present. Fluency denotes spatial and temporal flow in movement, paying particular attention to the velocity of movements. The performance domain relates to the achievement of motor milestones. The Total IMP score is a sum score based on the IMP domains (20). The IMP assessment provides a continuous score from 0 to 100 in each domain, with 100 indicating optimal motor function. The IMP domains adaptability and performance are age-dependent (20). This means that with increasing age and increased movement experience, the infant will learn to adapt its movements in the specific context and increase its motor skills. Hence, the scores corresponding to a given percentile value in these domains will increase by increasing age. As the total IMP score averages the scores from all five IMP domains, this score is also age-dependent (20). Psychometric properties of IMP are documented in several studies (24–27). In addition, two studies on predictive validity confirmed that IMP scores throughout infancy (assessed at 4, 10, and 18 months) were predictive of cognitive, behavioral, and neurological function at 4 and 9 years in a low-risk population (28, 29). A clinical study comparing two physiotherapeutic interventions suggested a clinically relevant change in IMP score to be 7.5 points (30).

The ASQ-2 is a worldwide used parent-reported instrument containing domains of gross and fine motor function, communication, personal and social skills, and problem-solving skills. Each item has questions about domain-specific activities, which are answered “yes” (10 points), “sometimes” (5 points), or “not yet” (0 points). Each domain provides a five-point interval score between 0 and 60, where 60 indicates optimal skills (21). The reliability and validity of the questionnaire are supported by evidence (21) and recommended for developmental screening by the American Academy of Pediatrics (AAP) (31). Due to the scope of this study, we only analyzed the parent reports on infant gross- and fine motor skills.

### Confounding Factors and Covariates

Based on findings from a previous study on maternal folic acid use in Norway (32) we included the following variables as potential confounding factors for the association of folic acid and multivitamin use with motor function; maternal age (<25, 25–34, >34 years), marital status (single/other, cohabitant, married) and parity (0, 1, ≥2). Other variables (not considered as confounding factors) included for description purposes only were maternal prepregnancy body mass index (BMI), gestational age (GA), infant birth weight, head circumference, and APGAR score at 5 min. GA was expressed as completed age of gestation in weeks and based on second-trimester ultrasound measurement, or if ultrasound data was missing, on the first day of the last menstrual period.

### Statistical Analysis

All analyses were performed using Stata IC version 16 (StataCorp, College Station, TX). The data on exposure (folic acid tablets or folic acid-containing multivitamins) from the MBRN was grouped as a categorical variable with three levels: 0: no use of either folic acid or multivitamin before or during pregnancy (reference category); 1: use of folic acid and/or multivitamin during pregnancy only; and 2: use before and during pregnancy (5). Women who reported supplement use before pregnancy only were placed in category 2. In an additional analysis, we incorporated supplement use as an ordered variable according to four increasing folic acid doses (i.e., no supplement use, multivitamin use alone, folic acid use alone, both folic acid and multivitamin use), with no use of folic acid or multivitamin supplements as the reference category. To estimate the association of exposure variables with each of the domains of IMP and the gross- and fine motor domains of ASQ-2, we used linear regression models. To account for unequal variability of the outcome variables across the range of supplement use groups, we further used robust variance estimation of regression coefficients. The associations are presented as crude and adjusted regression coefficient β (i.e., the difference in mean outcomes between exposure groups) with 95% confidence intervals (CI). Due to multiple testing of IMP and ASQ-2 domains and thus the probability of chance capitalization, the significance level was set at *p* < 0.01. Additionally, a sensitivity analysis excluding preterm infants was performed.

Due to the age-dependency in IMP total score and the IMP domains adaptability and performance, the different scores should be considered in relation to the age of the infants. As our sample has an age range from 3 to 18 months, it is relevant to also examine the age-specific association between folic acid-containing supplements and motor function. Hence, in a supplemental analysis, we divided our study sample into three age groups: 1: infants aged 3–6 months, 2: infants aged 7–12 months, and 3: infants aged 13–18 months. The associations of folic acid-containing supplements with IMP and ASQ-2 scores were estimated for each age group using linear regression models with robust standard error estimation.

## Results

Maternal and infant clinical characteristics are provided in Table 1. Of the 503 infants (261 boys and 242 girls) recruited in this study, 479 infants were term-born [mean gestational age for the total sample was 39.6 weeks and mean birth weight was 3,547 g (SD 531 g)]. About half of the infants (48.7%) were first born (i.e., parity 0). The number of infants per age group varied from *n* = 50 in the 7-month age group to *n* = 4 in the 13-month age group (the mean number of infants across age groups was 31.4). Only small differences in GA and corrected age at assessment were found between the exposure groups. One infant had missing data on ASQ-2 due to an incomplete questionnaire. The variables from the MBRN had some missing data (Table 1). Mean age for the mothers was 31 years (SD 4.5) and 34.4% were married.

**Table 1:** Characteristics of participating mothers and infants.

**Maternal data**	**Total no of subjects^**a**^** **(%)**	**No use of supplements** ***N* (%)**	**Supplement use during pregnancy only** ***N* (%)**	**Supplement use before and during pregnancy** ***N* (%)**
Maternal age at delivery				
<25 25–34 ≥35	26 (5.1) 323 (64.3) 154 (30.6)	8 (30.7) 73 (22.6) 31 (20.1)	10 (38.6) 138 (42.7) 76 (49.4)	8 (30.7) 112 (34.7) 47 (30.5)
Marital status				
Single/other Cohabitant Married	14 (2.8) 315 (62.8) 173 (34.4)	1 (7.1) 72 (22.8) 39 (22.5)	3 (21.4) 146 (46.2) 75 (43.4)	10 (71.5) 98 (31) 59 (34.1)
Prepregnancy BMI				
Underweight <18.5 Normalweight 18.5–24.9 Overweight 25–29.9 Obese ≥30	6 (1.3) 285 (61.9) 111 (24.2) 58 (12.6)	1 (16.6) 72 (25.3) 16 (14.4) 14 (24.1)	3 (50) 125 (43.8) 51 (45.9) 25 (43.1)	2 (33.4) 88 (30.9) 44 (39.7) 19 (32.8)
Parity				
0 1 ≥2	245 (48.7) 187 (37.2) 71 (14.1)	53 (21.6) 43 (23.1) 16 (22.5)	110 (44.9) 83 (44.4) 31 (43.7)	82 (33.5) 61 (32.5) 24 (33.8)
**Infant data**				
GA in weeks, mean (SD) [min-max]	39.6 (1.59)	39.5 (1.4) [33–42]	39.7 (1.6)	39.5 (1.7)
Corrected age at assessment mean (SD) [min-max]	8.5 (3.5)	8.3 (4.0) [3–18]	8.5 (3.7)	8.5 (4.2)
Birth weight, g (SD) [min-max]	3,547 (531)	3,476 (495) [1,560–5,240]	3,595 (526)	3,532 (559)
Head circumference, cm (SD) [min-max]	35 (1.4)	35 (1.3) [30–39]	35 (1.6)	35 (1.4)
APGAR 5 min (SD) [min-max]	9.5 (1)	9.5 (1) [1–10]	9.5 (0.9)	9.3 (1.1)

*Maternal age (in years), BMI Body Mass Index (kg/m^2^), Parity (number of previous births) GA gestational age, SD Standard deviation*.

a *Not all numbers in the column add up to the total sample (n = 503) due to missing data*.

In general, there was a high mean score, indicating a more or less optimal motor function for the majority of infants (Table 2). The term born infants had significantly higher scores in both gross and fine motor function as measured by ASQ-2 (*p* = 0.009 and *p* = 0.015, respectively). No difference between term-born and preterm infants was found for the IMP total and domain scores.

**Table 2:** Descriptive statistics of outcome variables.

**Outcome variable**	**All** **(*****n*** **=** **503)**		**No use of supplements** **(*****n*** **=** **112)**		**Use of folic acid and/or multivitamins during pregnancy only** **(*****n*** **=** **167)**		**Use of folic acid and/or multivitamins before and during pregnancy^b^** **(*****n*** **=** **224)**	
**IMP score (0–100)**	**Mean, (SD)**	**(Min-Max)**	**Mean, (SD)**	**(Min-Max)**	**Mean, (SD)**	**(Min-Max)**	**Mean, (SD)**	**(Min-Max)**
Variation	95.2, (5.9)	(69–100)	93.5, (5.8)	(75–100)	94.1, (6.2)	(69–100)	94.5, (5.9)	(70–100)
Adaptability	87.1, (9.1)	(63–100)	85.6, (9.4)	(64–100)	88.7, (8.9)	(63–100)	86.7, (9.0)	(67–100)
Fluency	96.7, (7.0)	(60–100)	97.7, (5.1)	(75–100)	96.9, (7.0)	(70–100)	96.2, (7.9)	(60–100)
Symmetry	99.4, (3.3)	(63–100)	99.7, (1.3)	(94–100)	99.3, (3.3)	(72–100)	99.2, (3.9)	(63–100)
Performance	74.4, (15.6)	(35–99)	74.1, (15.3)	(39–99)	73.7, 17.0	(36–98)	75.1, (14.7)	(35–97)
Total score	90.2, (5.5)	(64–99)	90.2, (4.9)	(74–99)	90.2, (5.9)	(75–99)	90.2, (5.9)	(64–99)
**ASQ-2 score (0–60)**								
Gross motor	47.6, (14.4)	(0–60)	49.6, (12.6)	(10–60)	47.4, (14.2)	(5–10)	46.7, (15.2)	(0–60)
Fine motor	50.9, (11.1)	(5–60)	51.6, (10.2)	(20–60)	49.8, (11.5)	(10–60)	51.5, (11.1)	(5–60)

*IMP Infant motor profile, ASQ-2 Ages and Stages questionnaire second edition. SD Standard deviation*.

*^a^The subdomain adaptability is only scored in infants >6 months according to the IMP manual*.

b *Women who reported using folic acid or multivitamins before pregnancy only was n = 8*.

Overall, 22.3% (*n* = 112) of the mothers did not use any folic acid-containing supplements before or during pregnancy; 33.2% (*n* = 167) initiated folic acid-containing supplements during pregnancy only, and 44.5% (*n* = 224) of mothers used folic acid-containing supplements both before and during pregnancy. Of the latter 224 women, eight women reported using folic acid-containing supplements before pregnancy only. The overall use of multivitamin tablets before and during pregnancy was 15.9 and 40.3%, respectively. The number of women using only multivitamins (and not folic acid) before and during pregnancy was 13.

### Folic Acid-Containing Supplement Use and IMP Scores

Overall, no significant associations were found between folic acid-containing supplement use and IMP domain scores in the total sample. When compared to no supplement use; infants born by mothers who reported folic acid-containing supplement use during pregnancy only, had higher scores on the domain adaptability (adjusted β = 2.87 95% CI = 0.08, 5.68) (Table 3). Further, when dividing the sample into age groups, the association was both stronger and significant in the infant age group 7–12 months (adjusted β = 4.41 95% CI = 1.12, 7.70), *p* = 0.01. No significant association was found for the infant age group 13–18 months (Supplementary Table 1).

**Table 3:** Associations of folic acid-containing supplement use with motor function.

	**Use of folic acid and/or multivitamins during pregnancy only**	**Use of folic acid and/or multivitamins before and during pregnancy^**a**^**
	**β** ^**b**^ **(95% CI)**	**β** ^**b**^ **(95% CI)**
IMP variation		
Crude Adjusted^c^	0.58 (−0.84, 2.01) 0.50 (−0.94, 1.95)	0.99 (−0.37, 2.34) 1.03 (−0.29, 2.37)
IMP adaptability		
Crude Adjusted^c^	3.11 (0.38, 5.84) 2.87 (0.08, 5.68)	1.13 (−1.42, 3.67) 1.15 (−1.47, 3.78)
IMP symmetry		
Crude Adjusted^c^	−0.36 (−1.15, 0.43) −0.39 (−0.97, 0.18)	−0.52 (−1.26, 0.23) −0.49 (−1.04, 0.07)
IMP fluency		
Crude Adjusted^c^	−0.89 (−2.58, 0.79) −0.79 (−2.24, 0.65)	−1.5 (−3.10, 0.09) −1.4 (−2.78, 0.00)
IMP performance		
Crude Adjusted^c^	−0.28 (−4.02, 3.47) −0.11 (−3.97, 3.76)	1.10 (−2.45, 4.65) 1.43 (−2.01, 4.89)
IMP total score		
Crude Adjusted^c^	0.09 (−1.23, 1.42) 0.11 (−1.19, 1.40)	0.10 (−1.15, 1.36) 0.22 (−0.95, 1.40)
ASQ-2 gross motor		
Crude Adjusted^c^	−2.28 (−5.72, 1.17) −2.32 (−5.54, 0.89)	−2.91 (−6.18, 0.35) −2.81 (−5.93, 0.31)
ASQ-2 fine motor		
Crude Adjusted^c^	−1.85 (−4.49, 0.81) −1.83 (−4.44, 0.79)	−0.01 (−2.62, 2.41) −0.06 (−2.45, 2.32)

*CI Confidence Intervals, IMP Infant Motor Profile, ASQ-2 Ages and Stages Questionnaire 2nd edition*.

a *Women who reported to use folic acid or multivitamin before pregnancy only was n = 8*.

b *Coefficients (β) are the difference between groups (use vs. Non-use)*.

c *Adjusted for maternal age, marital status, and parity*.

Regarding the remaining domains, no associations of supplement use with motor function were found. Sensitivity analysis excluding preterm infants (*n* = 24), did not affect the results of the regression analysis, nor did the strength of the associations change markedly when women were grouped by the dose of folic acid (Table 4). Further, no significant associations were found in separate analyses for the three age groups (Supplementary Table 1).

**Table 4:** Dose-response associations of folic acid use with motor function^#^.

	**Use of multivitamins only** ***n* = 13**	**Use of folic acid only** ***N* = 184**	**Use of both multivitamins and folic acid** ***n* = 194**
	**β** ^**b**^ **(95% CI)**	**β** ^**b**^ **(95% CI)**	
IMP variation			
Crude Adjusted^c^	2.55 (0.09, 5.02) 2.47 (0.08, 4.86)	1.10 (−0.29, 2.49) 1.11 (−0.30, 2.52)	0.42 (−0.94, 1.79) 0.42 (−0.95, 1.79)
IMP adaptability			
Crude Adjusted^c^	2.64 (−2.78, 8.07) 2.44 (−2.89, 7.79)	1.42 (−1.30, 4.15) 1.32 (−1.41, 4.05)	2.36 (−0.31, 5.04) 2.25 (−0.45, 4.96)
IMP symmetry			
Crude Adjusted^c^	−0.16 (−1.07, 0.73) −0.18 (−1.07, 0.72)	−0.40 (−0.99, 0.73) −0.39 (−1.01, 0.22)	−0.51 (−1.08, 0.05) −0.51 (−1.10, 0.07)
IMP fluency			
Crude Adjusted^c^	2.29 (1.34, 3.24)^*^ 2.21 (1.14, 3.28)	−1.30 (−2.77, 0.16) −1.18 (−2.65, 0.28)	−1.42 (−2.84, −0.00) −1.33 (−2.76, 0.09)
IMP performance			
Crude Adjusted^c^	5.26 (−3.15, 13.68) 5.08 (−3.20, 13.37)	1.20 (−2.40, 4.82) 1.64 (−1.99, 5.27)	−0.46 (−4.10, 3.17) −0.31 (−3.95, 3.34)
IMP total score			
Crude Adjusted^c^	2.38 (−0.08, 4.85) 2.30 (−0.05, 4.67)	0.24 (−0.97, 1.46) 0.35 (−0.88, 1.58)	−0.19 (−1.41, 1.02) −0.14 (−1.37, 1.09)
ASQ-2 gross motor			
Crude Adjusted^c^	−0.79 (−8.82, 7.22) −0.74 (−8.98, 7.50)	−2.38 (−5.55, 0.78) −2.32 (−5.52, 0.89)	−3.01 (−6.16, 0.14) −3.00 (−6.20, 0.19)
ASQ-2 fine motor			
Crude Adjusted^c^	1.85 (−3.67, 7.37) 1.72 (−3.79, 7.24)	−0.87 (−3.42, 1.67) −0.81 (−3.39, 1.76)	−1.01 (−3.46, 1.44) −0.97 (−3.42, 4.35)

*CI Confidence Intervals, IMP Infant Motor Profile, ASQ-2 Ages and Stages Questionnaire 2nd edition*.

b *Coefficients (β) are the difference between groups (use vs. Non-use)*.

c *Adjusted for maternal age, marital status, and parity*.

# *The number of mothers not using any supplements is 112*.

* *p = 0.01*.

### Folic Acid and Multivitamin Use and ASQ-2 Scores

No associations were found between folic acid-containing supplement use and the gross and fine motor domains of ASQ. Adjusting for maternal age, marital status, and parity did not influence the results, nor did the strength of the associations change markedly when women were grouped by the dose of folic (Table 4) or when infants were grouped by age in months (Supplementary Table 1). Sensitivity analysis excluding preterm infants (*n* = 24), did not affect the outcome of the regression analysis.

## Discussion

We examined whether the use of maternal folic acid-containing supplements before and during pregnancy affected motor function in infancy. In our study, we did not find any support for our predefined hypothesis about a positive association between folic acid-containing supplement use and increased motor function in offspring up to 18 months. We found a possible association between supplement use and the IMP domain adaptability (adjusted β = 2.88; 95% CI = 0.08, 5.68). This association remained strongest for infants aged 7–12 months, but the mean difference was below the clinically relevant difference (30). Further, the additional analysis of a dose-response association did not reveal any significant or clinically relevant differences. Our findings support the null findings from some of the previously mentioned studies (16, 17), but the evidence is mixed. The inconsistent findings are likely to be caused by differences in design, sample selection, and study size, statistical methods as well as different measures of exposure and assessment of motor function.

Motor function is not a fixed construct, and it can be problematic to compare the results of studies using different outcome measures. Previous epidemiological studies have utilized global developmental assessment batteries as outcome measures. Even though they are recommended as screening instruments (31) and parent reports have been found predictive of developmental delays (33), these instruments usually contain few items on gross and fine motor function. Consequently, minor impairments may not be detected. Hence, this may be a reason for discrepancies between studies investigating motor function. Although we know that motor trajectories between term-born and preterm infants are somewhat different (34), and the term-born infants in our sample had higher ASQ-2 scores, excluding preterm infants from our sample did not affect the outcome of the linear regression analysis.

Despite the good psychometric characteristics of the ASQ-2 and IMP (21, 24–27), the long-term implications of any relationship with folic acid-containing supplement use, are uncertain. Previous studies contained participants up to 5 years of age (14, 15, 17), while our sample only included infants up to 18 months. Hence, any possible later consequences could thus not be discovered. Although IMP shows good predictive validity (28, 29), instability in motor assessment scores can be found in both high-risk, and low-risk infants (35, 36). Hence, assessment of infants as young as 3 months at a single time point (as was the case in our study) may not be sufficient to detect “definite” minor motor difficulties. Future studies should, therefore, consider conducting endpoint assessments in older infants.

As to the statistical analysis, we checked normality of residuals for all regression models using histograms and Q-Q plots. The strongest deviations from the normal distribution were found for the IMP domain scores fluency and symmetry. In our population, nearly all infants had close to perfect scores of fluency and symmetry, leaving a strong ceiling effect for these outcomes. Accordingly, the estimated difference in fluency and symmetry between compared groups should be interpreted with caution.

A strength of our study is the prospective design providing accuracy of data collection regarding exposure, endpoints, and confounders. In contrast to case-control studies, the exposure status is collected ahead of the outcome, thus avoiding recall bias. Further, the prospective design also offers the possibility to examine several outcomes. In our study, we combined both parent reports of general development and a thorough clinical assessment of motor function. The scope of our study was motor function, but it could also be relevant to examine the other domains of ASQ-2 (communication, problem-solving skills, and personal and social skills) in relation to supplement use in a future study. Finally, a large number of variables from the MBRN were available. Accordingly, we were able to evaluate and account for several potential confounding variables. It is, however, also possible that our findings were influenced by unmeasured confounding factors. The relationship between maternal education and offspring motor function is inconclusive (37–39), but maternal education is recognized as a strong predictor of folic acid use, along with planned pregnancy (32) and maternal lifestyle (16, 40). Unfortunately, these variables are not registered in the MBRN but should be included in future research. We cannot know whether our findings would be different if we had access to these variables, but it would be relevant in order to describe the sample, thus enabling a comparison with other study samples.

Some limitations should be noted. The main concern is that we lack accurate measures of plasma folate concentration. Further, we acknowledge that not only folic acid, but a broad specter of nutrients is essential for the developing brain both during gestation and postpartum. In our study, the dose-response analysis did not reveal any differences, but the results could also be affected by the inaccuracy in exposure measurement. Hence, future studies should collect detailed data on maternal nutrition (preferably with blood samples), whether the infants were breastfed or bottle-fed, and timepoint for introducing solid food.

As the recruitment of participants was based on voluntary participation, it may challenge the representativeness of the sample. We had no data on the Non-participating mothers and infants, nor any data on why eligible parents decided not to participate in the study. Consequently, there may be a risk of self-selection bias as we do not know whether the individuals included differed in other relevant clinical characteristics or exposure from those who did not agree to participate. Even though our sample may be Non-representative for the general population, other studies in similar settings from Norway with a Non-representative cohort have shown that exposure-outcome associations do not necessarily lead to selection bias when compared to nationwide registry data (41, 42). Finally, the grouping of infants according to age may require a larger sample size than was the case in the present study. Future studies should thus consider to include infants within a narrower age range.

## Conclusion

Our study could not find any clinically relevant association of maternal folic acid-containing supplement use before or during pregnancy with offspring motor function. However, due to the mixed evidence, additional prospective studies on this relationship should be conducted. Future research would benefit from more comprehensive and accurate measures of exposure including a detailed maternal nutritional profile.

## Data Availability Statement

The raw data supporting the conclusions of this article will be available by the authors upon request.

## Ethics Statement

The studies involving human participants were reviewed and approved by Regional Committees for Medical and Health Research Ethics. Written informed consent to participate in this study was provided by the participants' legal guardian/next of kin.

## Author Contributions

KM contributed to the design, data collection, analysis, funding acquisition, wrote the initial draft of the manuscript, edited, and approved the final manuscript. RM contributed to the design of the study, the statistical analysis, editing of the manuscript, and approved the final manuscript. TD contributed to the design of the study, read, edited, and approved the final manuscript. All authors contributed to the article and approved the submitted version.

## Funding

This work was supported by the Norwegian Fund for Post-Graduate Training in Physiotherapy. The funding source had no involvement in study design, data collection, or interpretation of results.

## Conflict of Interest

The authors declare that the research was conducted in the absence of any commercial or financial relationships that could be construed as a potential conflict of interest.

## Publisher's Note

All claims expressed in this article are solely those of the authors and do not necessarily represent those of their affiliated organizations, or those of the publisher, the editors and the reviewers. Any product that may be evaluated in this article, or claim that may be made by its manufacturer, is not guaranteed or endorsed by the publisher.
