# Supplementary material for: Maternal Folic Acid-Containing Supplement Use in Relation to Offspring Motor Function. A Prospective Study of 503 Mother-Child Dyads
Source: Front Pediatr. 2022 Apr 5;10:789158. doi: 10.3389/fped.2022.789158 (PMC9016160; doi:10.3389/fped.2022.789158)
Supplement: Supplementary file 1 [file Table_1.pdf]

**Supplemental Table 1. Associations of folic acid use with motor function according to age groups #**

| | Use of folic acid and/or multivitamins during pregnancy only (n=167) | | Use of folic acid and/or multivitamins before and during pregnancy (n=224)[1] | |
|---|---|---|---|---|
| | Crude | Adjusted[3] | Crude | Adjusted[3] |
| Outcome | β [2] (95% CI) | β [2] (95% CI) | β [2] (95% CI) | β [2] (95% CI) |
| IMP variation | | | | |
| 3-6 months (n=229) | 0.59 (-1.7-2.89) | 0.64 (-1.66,2.95) | 2.45 (0.30,4.60)¤ | 2.68 (0.54,4.81)¤ |
| 7-11 months (n= 207) | 0.98 (-0.89,2.85) | 0.75 (-1.18,2.68) | -0.53 (-2.32,1.27) | -0.49 (-2.33,1.35) |
| 12-18 months (n= 67) | 0.31 (-3.29,3.92) | 0.37 (-3.46,4,21) | 0.58 (-2.98,4.14) | 0.65 (-3.61,4.92) |
| IMP adaptability[4] | | | | |
| 7-11 months (n= 207) | 4.14 (1.13,7.69)* | 4.41 (1.12,7.70)* | 0.93 (-2.17,4.04) | 0.93 (-2.13,3.03) |
| 12-18 months (n= 67) | 2.33 (-2.63,7.09) | 1.33 (-3.58,6.25) | 2.76 (-2.13,7.66) | 2.32 (-2.82,7.48) |
| IMP symmetry | | | | |
| 3-6 months (n=229) | -0.14 (-0.77,0.47) | -0.16 (-0.78,0.46) | -0.76 (-0.91,0.35) | -0.25 (-0.91,0.40) |
| 7-11 months (n= 207) | 0.13 (-0.12,0.39) | 0.10 (-0.15,0.36) | -0.25 (-0.87,0.37) | -0.29 (-0.97,0.38) |
| 12-18 months (n= 67) | -2.15 (-5.14,0.83) | -2.03 (-5.12,1.06) | -2.50 (-6.0,1.00) | -2.38 (-6.58,1.81) |
| IMP fluency | | | | |
| 3-6 months (n=229) | -1.14 (-3.94,1.14) | -1.23 (-3.75,1.29) | -2.39 (-4.88,0.09) | -2.34 (-4.81,0.11) |
| 7-11 months (n= 207) | 0.71 (-0.60,2.02) | 0.67 (-0.70,2.05) | -0.65 (-2.35,1.04) | -0.72 (-2.48,1.03) |
| 12-18 months (n= 67) | -2.89 (-6.34,0.54) | -2.58 (-6.33,1.16) | -2.89 (-6.33,0.54) | -1.80 (-4.89,2.52) |
| IMP performance | | | | |
| 3-6 months (n=229) | -1.71 (-5.48,2.06) | -1.69 (-5.05,2.10) | 1.33 (-2.15,4.83) | 1.63 (-1.97,5.25) |
| 7-11 months (n= 207) | 2.46 (-0.71,5.64) | 2.19 (-0.96,5.35) | 0.59 (-2.46,3.65) | 0.69 (-2.40,3.78) |
| 12-18 months (n= 67) | -0.22 (-3.08, 2.64) | -0.09 (-3.19,3,17) | 0.2 (-2.34,2.74) | 0.56 (-2.68,3.81) |
| IMP total score | | | | |
| 3-6 months (n=229) | -0.78 (-2.37,0.81) | -0.74 (-2.34,0.85) | 0.13 (-1.36, 1.63) | 0.27 (-1.23,1.78) |
| 7-11 months (n= 207) | 1.71 (0.45,2.97)¤ | 1.60 (0.31,2.89)¤ | 0.03 (-1.31,1.36) | 0.04 (-1.34,1.42) |
| 12-18 months (n= 67) | -0.53 (-2.98,1.92) | -0.62 (-3.18,1.94) | -0.18 (-2.66,2.29) | -1.10 (-2.99,2.77) |
| ASQ-2 gross motor | | | | |
| 3-6 months (n=229) | -3.60 (-7.74,0.53) | -3.34 (-7.55,0.85) | -2.16 (-5.88,1.55) | -2.25 (-6.09,1.58) |
| 7-11 months (n= 207) | -1.21 (-6.6,4.23) | -1.78 (-7.46,3.90) | -3.33 (-8.59,1.93) | -3.29 (-8.62,2.03) |
| 12-18 months (n= 67) | -1.98 (-11.65,7.75) | -2.34 (-11.25,6.57) | -2.71 (-12.84,7.40) | -2.62 (-12.95,7.71) |
| ASQ-2 fine motor | | | | |
| 3-6 months (n=229) | -2.29 (-6.41,1.82) | -1.95 (-6.11,2.19) | 1.81 (-2.05,5,69) | 2.19 (-1.71,6.09) |
| 7-11 months (n= 207) | -1.07 (-4.66,2.52) | -1.47 (-5.26,2.31) | -1.37 (-4.52,1.78) | -1.53 (-4.68,1.61) |
| 12-18 months (n= 67) | -0.97 (-7.75,5.80) | -4.22 (-11.51,3.06) | -3.85 (-10.64,2.92) | -5.65 (-12.39,1.08) |

CI Confidence Intervals, IMP Infant Motor Profile, ASQ-2 Ages and Stages Questionnaire 2nd edition
[1]Women who reported to use folic acid or multivitamin before pregnancy only was n=8.
[2]Coefficients (β) are difference between groups (use vs non-use).
[3]Adjusted for maternal age, marital status and parity.
[4] Adaptability is only assessed in infants >6 months
# The number of mothers not using any supplements is 112.

¤ p= 0.05
* *p*= 0.01.
